# Plasmon-enhanced parabolic nanostructures for broadband absorption in ultra-thin crystalline Si solar cells†

**DOI:** 10.1039/d3na00436h

**Published:** 2023-08-24

**Authors:** Yeasin Arafat Pritom, Dipayon Kumar Sikder, Sameia Zaman, Mainul Hossain

**Affiliations:** a Department of Electrical and Electronic Engineering, University of Dhaka Dhaka 1000 Bangladesh mainul.eee@du.ac.bd; b Department of Electrical and Electronic Engineering, Bangladesh University of Engineering and Technology Dhaka 1205 Bangladesh; c Department of Electrical Engineering and Computer Science, Massachusetts Institute of Technology Cambridge MA 02139 USA

## Abstract

Sub-wavelength plasmonic light trapping nanostructures are promising candidates for achieving enhanced broadband absorption in ultra-thin silicon (Si) solar cells. In this work, we use finite-difference time-domain (FDTD) simulations to demonstrate the light harvesting properties of periodic and parabola shaped Si nanostructures, decorated with metallic gold (Au) nanoparticles (NPs). The active medium of absorption is a 2 μm thick crystalline-silicon (c-Si), on top of which the parabolic nanotextures couple incident sunlight into guided modes. The parabola shape provides a graded refractive index profile and high diffraction efficiencies at higher order modes leading to excellent antireflection effects. The Au NPs scatter light into the Si layer and offer strong localized surface plasmon resonance (LSPR) resulting in broadband absorption with high conversion efficiency. For wavelengths (*λ*) ranging between 300 nm and 1600 nm, the structure is optimized for maximum absorption by adjusting the geometry and periodicity of the nanostructures and the size of the Au NPs. For parabola coated with 40 nm Au NPs, the average absorption enhancements are 7% (between *λ* = 300 nm and 1600 nm) and 28% (between *λ* = 800 nm and 1600 nm) when compared with bare parabola. Furthermore, device simulations show that the proposed solar cell can achieve a power conversion efficiency (PCE) as high as 21.39%, paving the way for the next generation of highly efficient, ultra-thin and low-cost Si solar cells.

## Abstract 2

Au nanoparticle coated parabolic nanostructures offer enhanced broadband absorption in ultra-thin c-Si solar cells. Results show 14.5% enhancement in absorbed power for 800 nm < *λ* < 1600 nm and 21.39% conversion efficiency, with only 2 μm thick c-Si.

## Graphical abstract

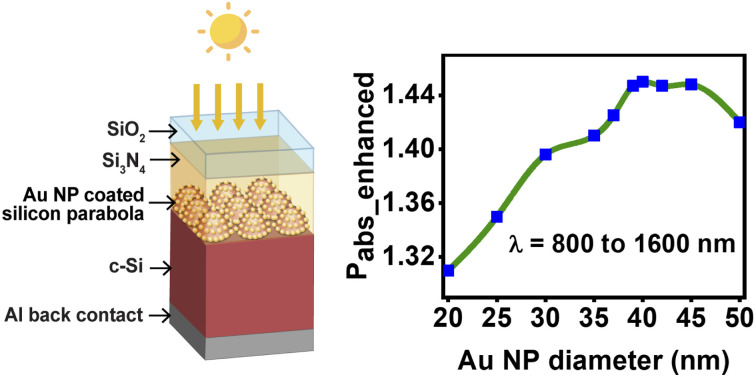

## 1. Introduction

Crystalline silicon (c-Si) solar cells have been widely commercialized due to their long-term stability, mature manufacturing process and the high natural abundance of Si. Realizing high efficiency c-Si solar cells requires hundreds of micrometres thick Si to absorb the maximum amount of the incident light. This, in turn, increases the material and production cost of Si solar cells.^1,2^ Ultra-thin c-Si solar cells have gained a lot of interest in recent years due to reduced material cost and potential for use in flexible and bendable photovoltaic devices.^3,4^ Unlike conventional wafer-based Si solar cells, which are 200–300 μm thick, ultra-thin film c-Si solar cells have an active layer which is only a few micrometres in thickness.^5^ The absorption loss due to reduced c-Si thickness is compensated by texturing the Si surface with sub-wavelength periodic nanostructures that minimize the front-surface reflection and enhance absorption by increasing the optical path length of the incident light through multiple scattering and interference effects.^6^ The size, geometry and orientation of the nanostructures can be carefully tuned to control the light absorption. For a randomly textured surface, at wavelengths near the band edge of Si, the maximum absorption enhancement, when averaged over all angles of incidence, is limited to 4*n*^2^ (Yablonovitch limit or the Lambertian limit) where *n* is the refractive index of Si.^7^ Previous studies have reported enhanced absorption in high-performance c-Si solar cells with various types of light-trapping Si nanostructures that include nanowires,^8^ nanopyramids,^9^ nanocones,^10^ paraboloids,^11^*etc.* However, the absorption coefficient of Si drops below 1000 cm^−1^ and approaches zero beyond the incident wavelength of 800 nm. This limits the absorption enhancement to the narrow spectral band of visible wavelengths only, resulting in the loss of a significant part of the incoming radiation.^12^ To achieve high efficiency c-Si solar cells, it is, therefore, necessary to enable enhanced broadband optical absorption in the long wavelength range of the solar spectrum.

The use of plasmonic metal nanoparticles (NPs) in organic, inorganic and hybrid solar cells has attracted much attention recently as they can provide enhanced absorption over a broad spectral range through large scattering cross-sections and near-field effects.^13–20^ Metal NPs, typically larger than 50 nm in diameter, act as subwavelength scatterers coupling the incident solar radiation into the active layer, thereby increasing the optical path length, and enhancing the absorption. However, ohmic losses in larger NPs limit their use. The near-field enhancements are brought about by the surface plasmons induced by the oscillating electric field of the incident radiation. Surface plasmons are defined as the collective oscillations of delocalized conduction band electrons at the surface of the metal NP and its surrounding dielectric medium. Along the metal–dielectric interface, surface plasmons can either propagate as electromagnetic waves known as surface plasmon polaritons (SPPs) or they can be confined around the NP giving rise to strong local field enhancements or localized surface plasmon resonance (LSPR). Near-field absorption enhancements through LSPR can be tailored by varying the type, geometry, size, and surrounding dielectric environment of the metal NPs.^21,22^ Additionally, metal NPs can increase optical absorption through non-radiative processes like hot electron transfer (HET) and plasmon resonant energy transfer (PRET).^23^ In HET, the strong near-fields surrounding the metal NPs induce intraband (within the conduction band) or interband transitions (d-band to sp band), which excite electrons (hot electrons) to a higher energy level above the Fermi level. Charge transmission results from these hot electrons which can tunnel through the Schottky barrier and get directly injected into the conduction band of neighbouring Si. In the PRET process, the absorbed solar energy is transferred from the metal NP to the Si absorber layer through dipole–dipole coupling. Here, the resulting electron–hole pairs, with energies below or near the bandgap of Si, are responsible for charge transmission.^22^

Several studies have demonstrated high optical absorption over a wide spectral range, followed by improved photocurrent and power conversion efficiencies (PCEs) in plasmon-enhanced ultra-thin Si solar cells.^24–28^ But most of the work is limited to planar devices and the use of plasmonic NPs to enable enhanced broadband absorption in textured Si solar cells has rarely been explored. Li *et al.*^29^ and Dawi *et al.*^30^ used finite-difference time-domain (FDTD) simulations to demonstrate GaAs nanowires decorated with gold (Au) NPs, where the particle size was tuned to obtain 50% and 35% absorption enhancements at 760 nm and 800 nm wavelengths, respectively. In our earlier work, we theoretically investigated plasmon enhancements in light-trapping Si nanowires, nanopyramids and flat-topped nanocones embedded with Au NPs and Si quantum dots.^31^

Superior light-trapping and broadband (350 nm to 1100 nm) anti-reflection properties of sub-wavelength parabolic Si nanostructures, compared to the widely used and high performing pyramid textures, have also been reported.^32,33^ Although pyramids are easy to fabricate and cost-effective, precise control of their geometrical dimensions is often difficult. Moreover, the height of pyramids at micron scale is challenging to implement in sub-50 μm ultra-thin Si wafers. On the contrary, shallow texturing of low-aspect ratio parabolic nanostructures offers a nearly linearly graded refractive index profile from the top to the bottom surface, thereby, minimizing reflection losses and concentrating the incident photons into the Si surface for enhanced absorption.^34,35^

This work is the first one to report the combination of Au NPs with parabolic nanostructures for enhanced light absorption in ultra-thin c-Si solar cells. We have used FDTD simulations to study the increase in optical absorption efficiency due to near field enhancements and far-field scattering events. The NPs efficiently scatter light into the absorber Si and the absorption enhancement induced by the LSPR effect from the plasmonic NPs can be enhanced by tailoring the size and distribution of the NPs. Moreover, the height (*H*) and base width (*W*_b_) of the paraboloid, along with its period (*P*), can also be tuned to minimize reflection losses and increase absorption efficiency. Finally, we performed device simulations to evaluate the performance of the proposed solar cell device.

## 2. Methodology

### 2.1 Device structure and simulation method

**Fig. 1:**
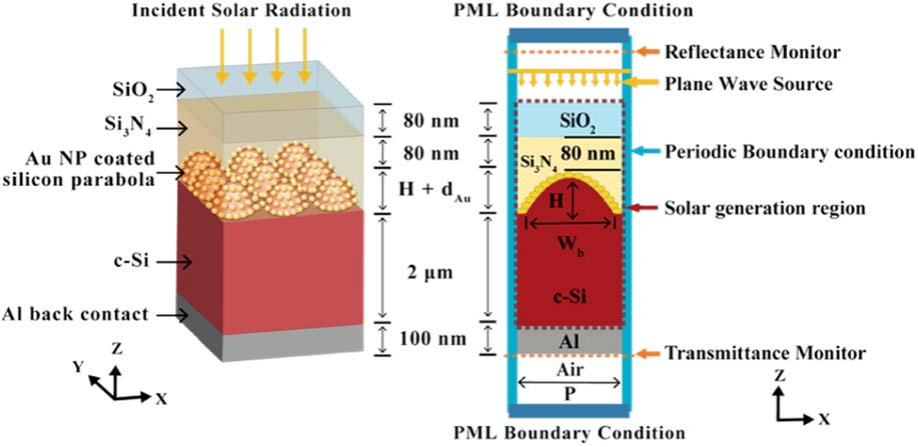
(a) 3D schematic of the c-Si solar cell with parabolic nanostructures decorated with gold nanoparticles (b) sectional view showing the FDTD simulation setup.

Fig. 1(a) shows a schematic of the proposed Si solar cell structure containing a three-dimensional (3D) array of parabolic nanostructures placed in a square lattice array. The paraboloids are decorated with Au NPs with a diameter of *d*_Au_. Plasmonic enhancement by metallic NPs depends on the surrounding material as well as the NP size, shape, and material. Gold is chosen due to its long-term stability and strong electric field confinement.^36^ The height (*H*), period (*P*) and the base width (*W*_b_) of the parabola are related by *H* = *P*^2^/8*R* where *R* is the radius of curvature of the parabola.^34^ The top surface, which is exposed to the incoming AM1.5G solar radiation at normal incidence, consists of an 80 nm thick anti-reflecting silica (SiO_2_) layer, followed by a 80 nm silicon nitride (Si_3_N_4_) and a 2 μm thick c-Si absorber layer. The thickness of the c-Si absorber layer is chosen as 2 μm in line with the previous studies on ultra-thin film c-Si solar cells.^37,38^ The active c-Si layer is textured with a periodic arrangement of parabola-shaped Si nanostructures, with height, *H* = 500 nm, whose sidewalls are decorated with Au NPs. The aluminium (Al) bottom contact or back electrode has a thickness of 100 nm. The antireflection and light trapping properties of the parabolic structures, with and without the embedded NPs, have been investigated using the commercially available Ansys Lumerical FDTD simulation package. The FDTD method solves Maxwell's equations to compute the electric and magnetic fields at every point of space and time in the solar cell structure, yielding wavelength dependent reflection *R*(*λ*), absorption *A*(*λ*) and transmission *T*(*λ*) spectra.

Fig. 1(b) illustrates the cross-sectional view of the simulated unit cell structure. A plane wave source, with wavelengths ranging between 300–1600 nm, irradiates the solar cell at normal incidence. All simulations are done with polarization angles set to 0° for a TM incident wave. This is because previous studies have shown stronger plasmonic excitations in the TM mode providing higher absorption enhancements, leading to higher carrier generation rate and higher short circuit current density in plasmon enhanced solar cells.^39–41^ To delineate this difference between TM and TE modes, the electric field patterns are evaluated for both the modes where the polarization angles for the TE incident wave are set to 90°. The electric field profiles are obtained by placing a two-dimensional field and power monitor in the *xz* plane through the centre of the parabola unit cell (Fig. S1†). Periodic boundary conditions were applied in the *x* and *y* directions which allows the periodic array to be modelled by carrying out simulations within the unit cell. Any reflected or transmitted fields were absorbed by implementing perfectly matched layer (PML) boundary conditions on the top and the bottom surfaces along the *z* direction. The absorbance *A*(*λ*) of the incident light can be calculated from the reflectance *R*(*λ*) and transmittance *T*(*λ*) values as *A*(*λ*) = 1 − *R*(*λ*) − *T*(*λ*). In the presence of the back-reflector, *T*(*λ*) ≈ 0 and therefore, *A*(*λ*) = 1 − *R*(*λ*). For an equally spaced wavelength interval (*dλ*), the average absorption (*A*_avg_) is obtained as:^34^1
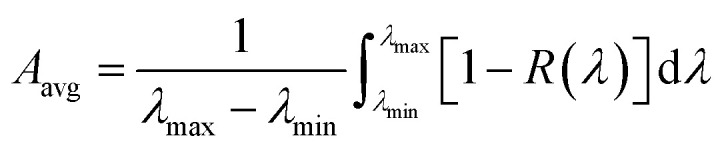


To obtain the power *P*_abs_(*λ*), absorbed by the active c-Si layer, a volume integration is performed over the entire volume (*V*) of the active c-Si region such that:^28^2
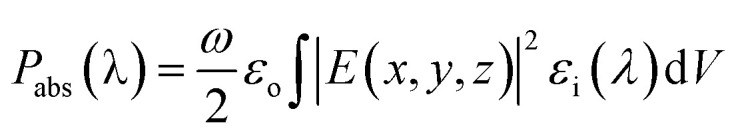
where, *E* is the time-averaged electric field within the absorber layer, *ω* is the angular frequency of the incident light, *ε*_o_ denotes the permittivity of free space and *ε*_i_ is the imaginary part of the dielectric function in the active region. The absorbed power within the parabola is responsible for generating the carriers in the active region and the corresponding absorption *A*(*λ*) can be normalized by considering the ratio of *P*_abs_(*λ*) to *P*_in_(*λ*) where *P*_in_(*λ*) is the power incident from the plane wave source:^28^3
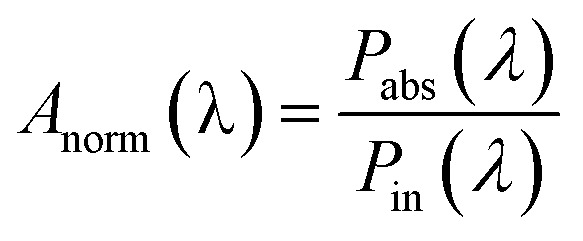


The enhancement in absorbed power (*P*_abs_enhanced_) due to the incorporation of the Au NPs can be computed by taking the ratio of the absorbed power with and without the Au NPs as:^42^4
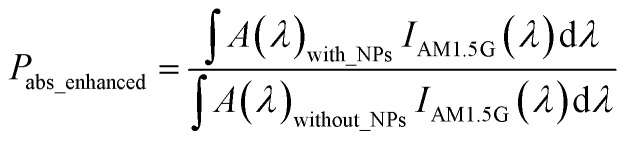
where, *A*(*λ*)_with_NPs_ and *A*(*λ*)_without_NPs_ are the absorbed powers in c-Si, with and without the Au NPs at wavelength *λ*, and *I*_AM1.5G_(*λ*) is the spectral irradiance of the incident AM1.5G solar radiation.

Assuming all photogenerated carriers contribute to the photocurrent, the resulting short-circuit current density (*J*_SC_) of the solar cell is obtained by:^28^5
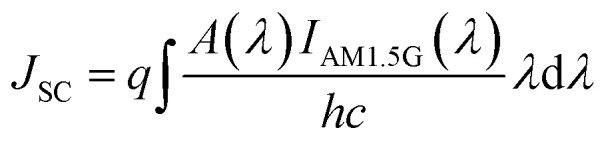
where, *q* is the electronic charge, *c* is the speed of light and *h* is the Planck's constant. Finally, the conversion efficiency (*η*) of the plasmon enhanced solar cell is calculated as follows:^31,43^6
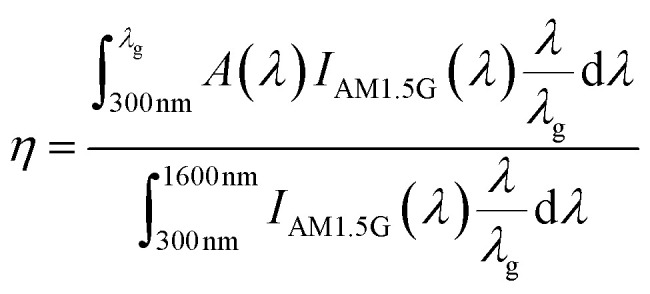
where, *λ*_g_ = 1127 nm is the wavelength corresponding to the bandgap (1.12 eV) of Si. The relation assumes that each absorbed photon, with energy greater than the bandgap, produces one and only one electron–hole pair with energy *hc*/*λ*_g_. The upper (1600 nm) and lower (300 nm) wavelength limits of the integral, in the denominator, are chosen to cover the entire range of the solar spectrum used in the simulations. Finally, the charge generation profile obtained from the FDTD simulations is fed into Lumerical-CHARGE module to compute the electrical characteristics of the proposed solar cell device, considering Auger, trap-assisted, radiative, and surface recombinations. A similar approach has been adopted in literature where a 3D charge profile from FDTD simulations accurately predicts the electrical characteristics in one-dimension (1D).^44,48^Table 1 lists the input parameters used in the electrical simulations.

**Table 1:** Electrical parameters used in device simulations^44–47^

Parameters	Description	Nominal values
*μ*_n_	Electron mobility	1471 cm^2^ V^−1^ s^−1^
*μ*_p_	Hole mobility	470.5 cm^2^ V^−1^ s^−1^
*N*_A_	Acceptor P doping	1 × 10^16^–1 × 10^18^ cm^−3^
*N*_D_	Donor N doping	1 × 10^16^–1 × 10^18^ cm^−3^
*N*_S_	Substrate P^+^ doping	1 × 10^19^ cm^−3^
*τ*_n_	Electron SRH recombination lifetime	90 ns–7 μs
*τ*_p_	Hole SRH recombination lifetime	30 ns–9 μs
*C*_n, Auger_	Auger recombination of electron at 300 K for crystalline Si	2.8 × 10^−31^ cm^6^ s^−1^
*C*_p, Auger_	Auger recombination of hole at 300 K for crystalline Si	9.9 × 10^−32^ cm^6^ s^−1^
*C*_radiative_	Radiative recombination at 300 K for crystalline silicon	1.6 × 10^−14^ cm^6^ s^−1^
SRV	Surface recombination velocity	10^3^–10^7^ cm s^−1^
*m*_n_ a	Effective mass of electron in crystalline Si at 300 K	1.18*m*_o_
*m*_h_ a	Effective mass of hole in crystalline Si at 300 K	0.8098*m*_o_
*ε*_r_	Relative permittivity for crystalline Si	11.7
*E*_g_	Bandgap	1.12 eV

a Used for denoting effective masses; *m*_o_ = rest mass of electron in crystalline Si.

### 2.2 Proposed fabrication process

**Fig. 2:**
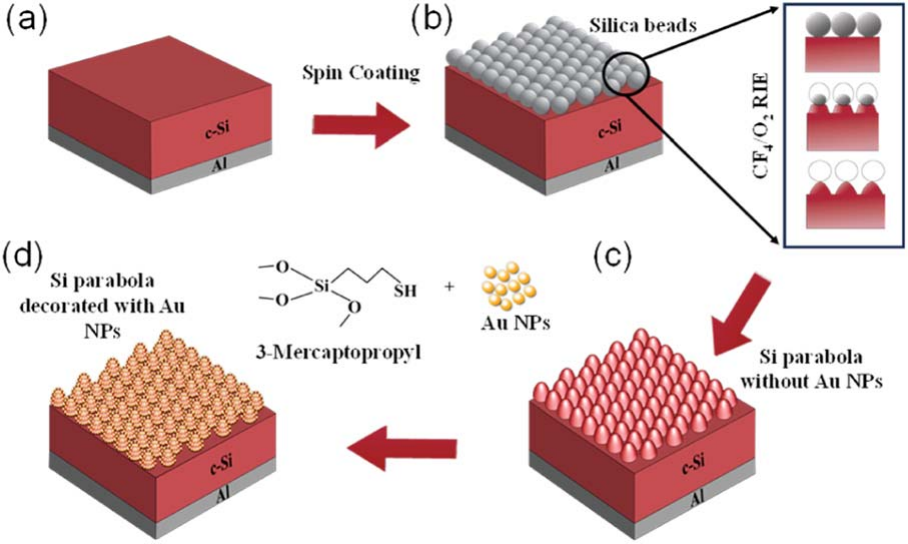
Proposed fabrication scheme for Si parabolic nanostructures decorated with Au NPs: (a) c-Si substrate with Al back contact (b) spherical silica beads spin-coated on top of c-Si. Inset shows the reactive-ion etching (RIE) process (c) c-Si solar cell with parabolic nanotextures functionalized with Si hydroxy group (d) conjugation with Au NPs in presence of 3-Mercaptopropyl-trimethoxysilane (MPTS) to form Au NP decorated parabolic nanostructures.

The proposed parabola shaped Si nanostructures can be fabricated by a combination of nanosphere lithography and reactive ion etching (RIE) process, as demonstrated by Cheon *et al.*^32^Fig. 2 summarizes the steps involved in the fabrication of Si parabolas, followed by the incorporation of Au NPs. Silica colloidal solutions containing spherical silica beads of different diameters are prepared and spin coated onto clean Si substrates. Silica self-assembled monolayers (SAMs) are formed, which are then used as dry etch masks in the RIE process. The SAMs are etched to completely remove the silica beads, allowing precise control of the height and shape of the fabricated Si parabola. Any Si beads remaining after the etching process are removed by rinsing the Si substrates in hydrofluoric acid (HF) solution. Next, the Si parabolas are coated with Au NPs, following the process described by Lin *et al.*^49^ First, the Si parabolas are functionalized with a Si hydroxy group and then immersed, first, in a solution containing 3-Mercaptopropyl-trimethoxysilane (MPTS) and then in a solution containing the Au NPs, followed by rinsing with water and exposure to UV light to remove the MPTS.

## 3. Result and discussion

The light trapping and anti-reflection properties of the paraboloids are largely governed by their geometry. Therefore, the base width (*W*_b_), height (*H*) and periodicity (*P*) of parabolic nanostructures are first optimized to achieve the best optical absorption properties.

### 3.1 Parabola geometry

**Fig. 3:**
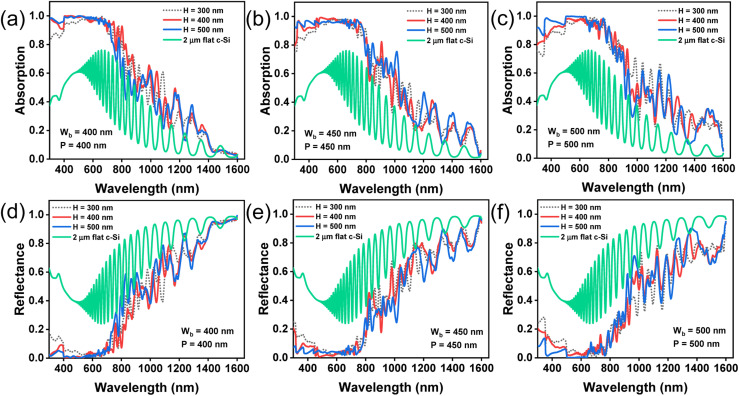
Results of FDTD simulations with TM polarized incident light showing absorption (a–c) and reflectance (d–f) spectra for different heights (*H* = 300 nm, 400 nm, and 500 nm) of bare parabolic nanostructures when the base width (*W*_b_) and period (*P*) are varied, keeping *W*_b_/*P* = 1.0. The absorption and reflection spectra of bare 2 μm thick c-Si slab is shown as a reference.

Fig. 3(a)–(c) show the absorption spectra of bare paraboloids (without any NPs) for different values of *H*, *W*_b_, and *P*. The filling ratio (*W*_b_/*P*) is kept fixed at 1.0. The absorption spectra for a flat 2 μm thick c-Si slab, without any texture, is shown as the reference. The oscillations observed in the long wavelength region are owing to the presence of Fabry–Perot resonance modes.^43^ The corresponding reflectance curves are shown in Fig. 3(d)–(f). Beyond the bandgap wavelength of Si (*λ*_g_ = 1127 nm), most of the incident light is transmitted through air in between the nanostructures, thereby, decreasing the absorption and increasing reflection losses. It is observed that at any given *W*_b_, the absorption increases with *H*. This can be attributed to the strong light trapping properties of high aspect ratio nanostructures, irrespective of the nanostructure shape. The incident light interferes with light diffracted from the nanostructures and undergoes a phase change. The phase change at any given wavelength depends on the height of the structure, which can be optimized to produce destructive interference conductions and yield minimum transmission field at the zeroth order.^34^ Increasing *H* beyond 500 nm may further enhance the absorption, but can also lead to increased surface recombination, thereby degrading the solar cell performance. Thus, *H* is kept fixed at 500 nm for the rest of this study.

**Fig. 4:**
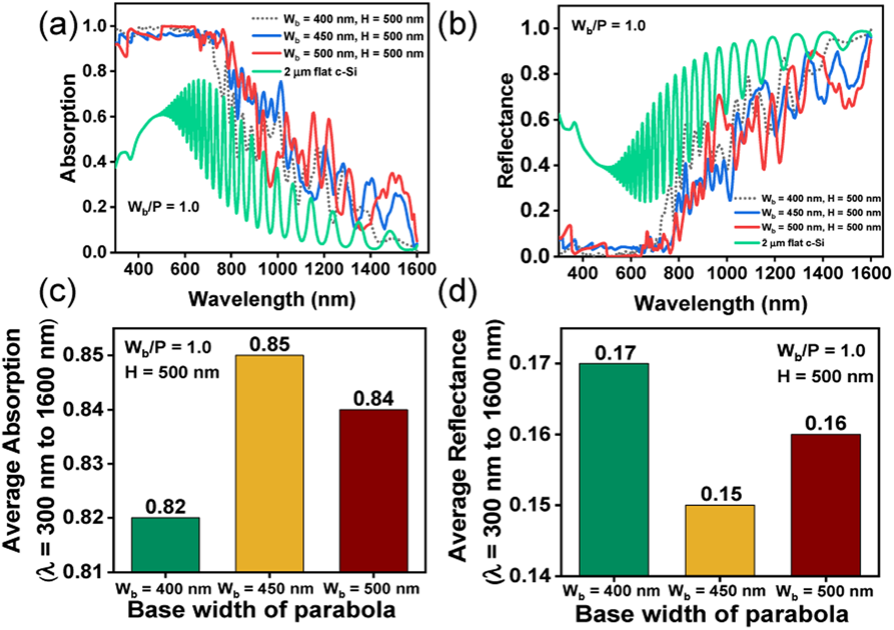
Results of FDTD simulations with TM polarized incident light showing (a) absorption and (b) reflectance spectra for bare parabola with different base widths (*W*_b_) keeping *H* = 500 nm and *W*_b_/*P* = 1.0; corresponding average (c) absorption and (d) reflectance, between *λ* = 300 nm and 1600 nm.

Fig. 4(a) and (b) illustrate the absorption and reflectance spectra, respectively, at three different values of *W*_b_, with *H* = 500 nm. The corresponding bar plots showing average absorption and reflection are displayed in Fig. 4(c) and (d), respectively. The results can be explained by considering the coupling of the incident light with guided resonance modes. In all cases, maximum absorption occurs in the visible part of the spectrum, beyond which the absorption degrades significantly. This is because at longer wavelengths, Si inherently exhibits low absorption coefficient and most of the incident photons are reflected into the air, without effectively inducing guided resonant modes within the nanostructures. Paraboloids with a larger *W*_b_ can support a larger number of resonant modes and therefore optical absorption, in general, increases (*i.e.* reflection decreases) with increasing *W*_b_.^44^ For *H* = 500 nm, the average absorption, between *λ* = 300 nm and 1600 nm, is maximum at *W*_b_ = 450 nm, as shown in Fig. 4(c). Increasing *W*_b_ to 500 nm increases reflection losses, as portrayed in Fig. 4(d), and reduces the concentration of supported resonant modes within the structure, leading to poor optical absorption efficiency.^50^ For further analysis in this study, *W*_b_ and *H* are fixed at 450 nm and 500 nm, respectively.

### 3.2 Periodicity of the parabolic nanostructures

**Fig. 5:**
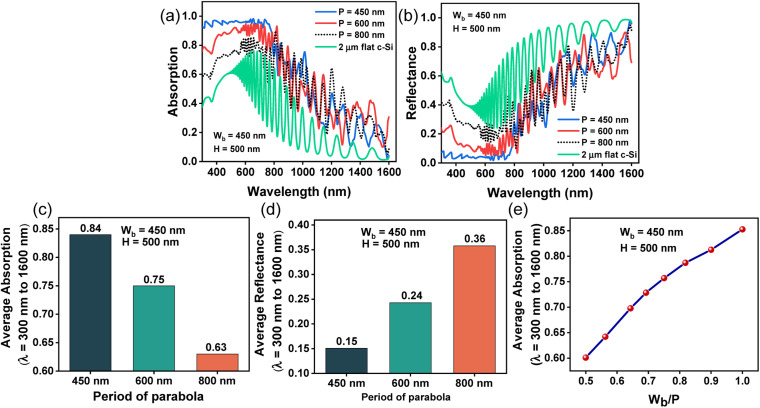
Results of FDTD simulations with TM polarized incident light showing the (a) absorption (b) reflectance spectra (c) average absorption (d) average reflectance and (e) the average absorption *vs. W*_b_/*P* ratio between *λ* = 300 nm and 1600 nm, for bare parabolic nanostructures having *W*_b_ = 450 nm, *H* = 500 nm and *P* = 450 nm, 600 nm, and 800 nm.

Fig. 5(a) and (b) illustrate the absorption and reflectance profiles, respectively, of bare parabolic surface textures having *W*_b_ = 450 nm, *H* = 500 nm, and period *P* = 450 nm, 600 nm, and 800 nm. The absorption spectra of a 2 μm thick planar Si slab is shown as the reference. Fig. 5(c) and (d) show the average absorption and reflectance, respectively, for variable periods, between *λ* = 300 nm and 1600 nm. Smaller periods exhibit maximum absorption (lowest reflection) towards shorter wavelengths while the absorption towards longer wavelengths increases with increasing period. Light is coupled more efficiently to the parabolic grating structure when the period of the paraboloids is slightly smaller than the incident wavelength. Periods which are much smaller than the interacting wavelength, lead to poor optical diffraction while larger periods weaken the absorption due to the presence of higher order reflection channels.^51^

Periodicity (*P*) is inversely related to the filling ratio.^52^ For a fixed *W*_b_, decreasing *P* increases *W*_b_/*P* and consequently enhances the average absorption between *λ* = 300 nm and 1600 nm, reaching its maximum value when *W*_b_ equals *P*, as displayed in Fig. 5(e). When the filling ratio (*W*_b_/*P*) < 1, *P* becomes too large, and the optical diffraction becomes weak, resulting in reduced absorption. Finally, based on the results obtained so far, the optimized parabola textures used for the rest of this study have a *W*_b_, *P* and *H* of 450 nm, 450 nm, and 500 nm, respectively.

### 3.3 Nanoparticle size

**Fig. 6:**
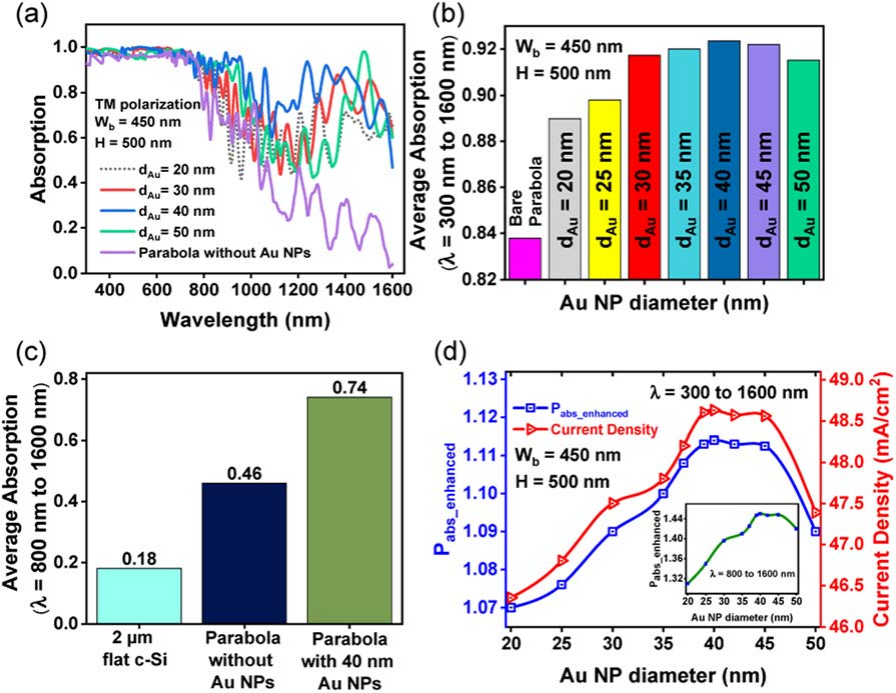
Results of FDTD simulations with TM polarized incident light showing (a) absorption spectra and (b) the average absorption between *λ* = 300 nm and 1600 nm for parabolic nanostructures coated with Au NPs of varying diameters; bare parabola is shown as a reference in each case (c) the average absorption between *λ* = 800 nm and 1600 nm for 2 μm thick bare Si slab, bare Si parabola and parabola coated with 40 nm Au nanoparticles (d) enhanced power absorption and current density of the solar cell, between *λ* = 300 nm and 1600 nm, for Au NPs having different diameters. Inset shows enhanced power absorption *vs.* Au NP diameter between *λ* = 800 nm and 1600 nm.

For the parabolic nanostructures, decorated with metallic NPs, light absorption is influenced by the size and shape of the NPs, the spacing of the NPs, the material of the NPs and the optical constants of the medium surrounding the NPs. Fig. 6(a) shows the absorption spectra of optimized parabolic nanotextures (*W*_b_ = 450 nm and *H* = 500 nm) decorated with spherical Au NPs, placed next to each other, and covering the entire surface of the parabola. The diameters (*d*_Au_) of the Au nanoparticles are taken as 20 nm, 30 nm, 40 nm, and 50 nm. The absorption *vs.* wavelength profile of the bare parabolic nanotextures, without the NPs, is shown as the reference. The incorporation of Au NPs significantly increases the absorption of light over a broad spectral range, leading to a stronger photocurrent response.

Fig. 6(b) illustrates the average absorption, between *λ* = 300 nm and 1600 nm, as a function of the Au NP diameter. Maximum absorption is obtained when *d*_Au_ = 40 nm. Compared to bare paraboloids, the Au NP incorporated paraboloids show a ∼7% increase in absorption, averaged over the entire wavelength range. For the parabola nanostructures, coated with 40 nm Au NPs, the average absorption between *λ* = 800 nm and 1600 nm is enhanced by 28% and 56% with respect to bare Si parabola and 2 μm thick bare Si, respectively, as shown in Fig. 6(c).

The enhancement in absorbed power (*P*_abs___enhanced_) between *λ* = 300 nm and 1600 nm, with change in NP diameter is plotted in Fig. 6(d). The filling ratio for each NP size is displayed in Table S1.† When integrated over the entire wavelength range (*λ* = 300 nm to 1600 nm) parabolic nanotextures decorated with 40 nm Au nanoparticles provides a maximum *P*_abs_enhancement_ of 1.114 (11.14%). The enhancement is more obvious (14.5%) between *λ* = 800 nm to 1600 nm, as shown in the inset of Fig. 6(d). The size dependent plasmonic enhancements obtained from the Au NPs can be explained as follows. Incident light is either coupled or forward scattered into the adjacent Si region by the Au NPs, resulting in multiple LSPR modes excited at different wavelengths. Smaller NPs with diameters ranging between 5–20 nm act as sub-wavelength antennas, coupling the incident field into the adjacent photoactive c-Si layer. However, surface scattering causes plasma damping in smaller NPs which limits the absorption enhancements. The size dependent scattering cross-sections of the Au NP embedded Si parabola (Fig. S2†) are obtained from Mie theory for different Au NP diameters, and are found to be consistent with results reported in literature.^53^ Moreover, broadband enhancement is not achieved by smaller NPs because of the narrow plasmon resonance range.^42^ Larger NPs, on the other hand, provide large scattering cross-sections, leading to an increase in optical path length and directing more light into the absorbing c-Si.^20,21,54^ Additionally, dynamic depolarization in larger NPs causes the plasmon resonance peak to be red shifted towards longer wavelengths and enables broadband absorption through radiation damping effects.^42^ For any given particle size, the average absorption also increases with the number of Au NPs per unit cell (Fig. S3†).

Fig. 6(d) also shows how the resulting current density (*J*_sc_) of the proposed solar cell device changes with NP size, where *J*_sc_ is obtained from eqn (5) using the absorption spectra in Fig. 6(a). The highest current density is obtained when *d*_Au_ = 40 nm, which also corresponds to maximum absorption enhancements through LSPR effects and scattering in the long wavelength regions. Also, the change in *J*_sc_ with NP size is in line with the light absorption efficiency (LAE)^55^ calculated for different NP diameters (Fig. S4†). For NPs larger than 40 nm in size, increase in absorption is associated with significant ohmic losses in the Au NPs, making near field effects impractical and resulting in reduced current density.^56^ The results are consistent with those reported by Zeng *et al.*^57^ and Piralaee *et al.*^58^

**Fig. 7:**
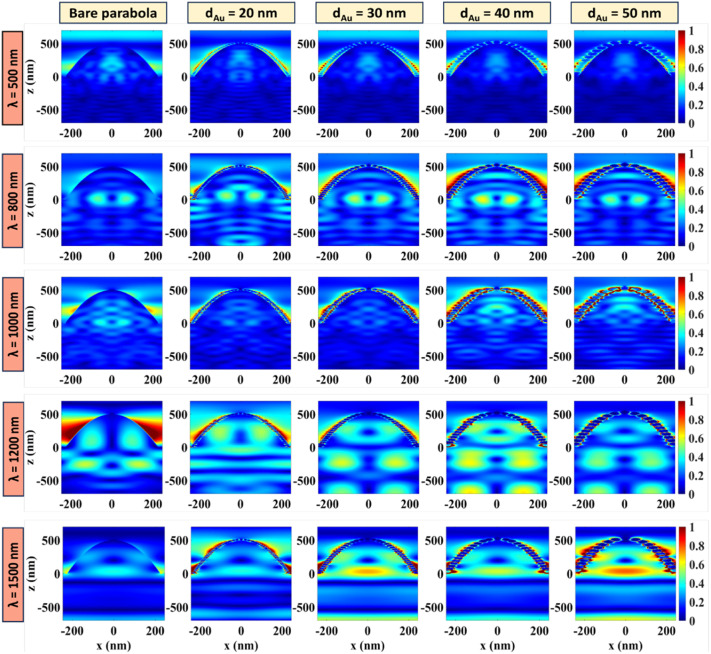
The normalized electric-field distributions of the bare and Au NP coated parabolic nanostructures for the TM polarized incident light at *λ* = 500 nm, 800 nm, 1000 nm, 1200 nm, and 1500 nm. The diameters of the Au NPs are taken as 20 nm, 30 nm, 40 nm, and 50 nm and bare Si parabola is shown as the reference.

To further understand how the plasmon-enhanced absorption changes with NP size, the distribution of the electric field vector, TM polarized along the *XZ* plane (*Y* = 0) of the parabola, is plotted in Fig. 7 at *λ* = 500 nm, 800 nm, 1000 nm, 1200 nm, and 1500 nm for *d*_Au_ = 20 nm, 30 nm, 40 nm, and 50 nm. In each case, the electric field confined inside a bare parabola (without any NPs) is taken as the reference. The colour bars on the right indicate the electric field intensity normalized with respect to the maximum value. When compared with the corresponding electric field plots (Fig. S5†) for the TE polarized incident light, it is clear that the TM polarized modes provide stronger absorption enhancements, as reported in literature.^39–41^ For smaller NPs, lower-order localized surface plasmon modes contribute to the trapped electric field whereas higher-order modes are excited by the larger NPs, coupling more light into the underlying c-Si absorber layer.^30^ Simulation results show maximum electric field confinement around the near bandgap wavelength of *λ* = 1200 nm, when *d*_Au_ = 40 nm, which is consistent with the absorption enhancement results displayed in Fig. 6. It is obvious that, at *λ* = 500 nm, the distribution of the electric field within the parabola remains almost unaffected after the incorporation of Au NPs, suggesting that the NPs at short wavelengths do not contribute much to the absorption enhancement. This can be attributed to the low extinction coefficient of the localized surface plasmons excited in the near-infrared spectrum. At longer wavelengths beyond the visible range, the electric field is highly concentrated around the Au NPs, allowing a significant portion of the incident light to be coupled into the neighbouring Si, compared to the bare parabola. The electric-field distribution for the 50 nm Au NPs at *λ* = 1500 nm show a stronger absorption profile than the 40 nm particles at the same wavelength. This is because of the sharp absorption peak observed at *λ* = 1500 nm for the 50 nm particle (Fig. 6(a)). Nevertheless, when averaged over the entire wavelength range, the 40 nm particles show maximum absorption (Fig. 6(b)). The results are consistent with the ones demonstrated by Li *et al.*^29^ and Dawi *et al.*^30^ for GaAs nanowire solar cells decorated with Au NPs.

### 3.4 Device characteristics

**Fig. 8:**
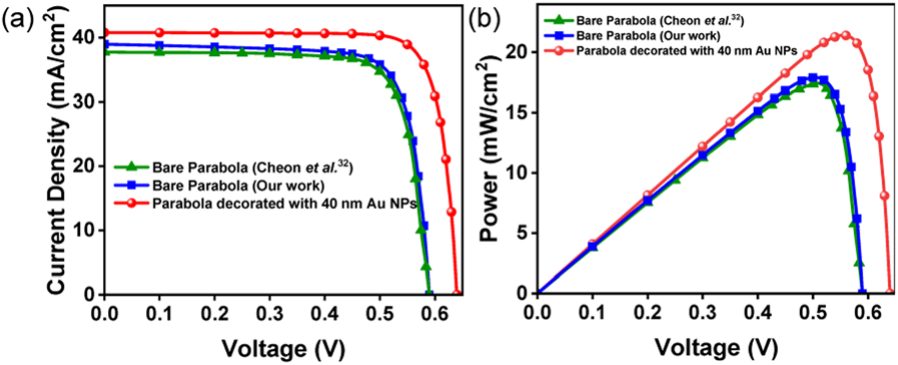
Results of device simulations showing (a) current *vs.* voltage (*J*–*V*) and (b) power *vs.* voltage (*P*–*V*) characteristics of bare parabolic nanotextures and parabolic nanostructures coated with 40 nm Au NPs. The electrical characteristics of the bare parabolic nanostructures fabricated by Cheon *et al.*^32^ is shown as a reference.

The current–voltage (*J*–*V*) and power–voltage (*P*–*V*) characteristics of the proposed solar cell device, with and without the 40 nm Au NPs are shown in Fig. 8(a) and (b), respectively. For the device with bare parabolic Si nanotextures, the simulated electrical characteristics match very well with the experimental results reported by Cheon *et al.*^32^Table 2 summarizes the output parameters of the solar cells. Results show that the incorporation of Au NPs enhances the *J*_sc_ and PCE by 4.73% (from 38.94 mA cm^−2^ to 40.78 mA cm^−2^) and 3.49% (from 17.90% to 21.39%), respectively. The performance of the proposed solar cell device is, therefore, comparable to or better than some of the other single junction plasmonic solar cells reported in literature.^5,37,59,60^

**Table 2:** Geometrical and photovoltaic output parameters of c-Si solar cells having bare parabolic nanotextures and parabolic nanostructures decorated with 40 nm Au NPs

Structure of the solar cell	Geometrical parameters	*J*_sc_ (mA cm^−2^)	*V*_oc_ (V)	FF (%)	PCE (%)
Bare parabolic textures without any NPs	*W*_b_ = 450 nm, *P* = 450 nm, *H* = 500 nm	38.94	0.59	77.91	17.90
Parabolic textures decorated with Au NPs	*W*_b_ = 450 nm, *P* = 530 nm, *H* = 500 nm, *d*_Au_ = 40 nm	40.78	0.64	81.96	21.39

## 4. Conclusion

We have used FDTD simulations to investigate the light harvesting properties of Si parabolic nanotextures coated with Au NPs as a potential candidate for highly efficient and ultra-thin plasmon-enhanced c-Si solar cells. The parabolic profile is chosen because it offers gradient refractive index from the incident to the absorbing medium, allowing a significant portion of the incident light to be diffracted into higher order modes, leading to enhanced absorption within the active Si layer. Additionally, the combination of near-field LSPR effects and far-field scattering from the Au NPs enables broadband absorption of longer wavelength photons by extending the absorption wavelengths beyond the visible spectrum. The geometry and periodicity of the parabola and the size of the NPs are optimized to achieve a maximum of 7% enhancement in light absorption, when averaged over the wavelengths ranging from *λ* = 300 nm to 1600 nm. This translates to 28% average absorption enhancement between *λ* = 800 nm to 1600 nm. Device simulations performed on the fully optimized plasmon-enhanced solar cell, with 2 μm thick active c-Si layer, demonstrate a PCE of 21.39%. Using well-established fabrication techniques, the plasmon enhanced parabolic nanostructures, proposed in the current simulation study, can be easily realized. We anticipate that the numerical results presented here can lead to new avenues for the design and implementation of ultra-thin, highly efficient, and low-cost c-Si solar cells enabled by plasmon-enhanced light trapping parabolic nanostructures.

## Author contributions

Y. A. Pritom and D. K. Sikder: performed all FDTD and device simulations, carried out data curation, formal analysis, review, and editing; Y. A. Pritom addressed the comments from the reviewers during revision. S. Zaman: performed validation and review; M. Hossain: conceptualized and supervised the entire project, performed formal analysis and validation, and wrote the original article. All authors have read and agreed to the published version of the manuscript.

## Conflicts of interest

There are no conflicts to declare.

## Supplementary Material

NA-005-D3NA00436H-s001
