# Supplementary material for: Plasmon-enhanced parabolic nanostructures for broadband absorption in ultra-thin crystalline Si solar cells
Source: Nanoscale Adv. 2023 Aug 24;5(18):4986–95. doi: 10.1039/d3na00436h (PMC10496899; doi:10.1039/d3na00436h)
Supplement: NA-005-D3NA00436H-s001 [file NA-005-D3NA00436H-s001.pdf]

Electronic Supplementary Material (ESI) for Nanoscale Advances.
This journal is © The Royal Society of Chemistry 2023

Electronic Supplementary Information

Plasmon-enhanced parabolic nanostructures for broadband absorption in ultra-thin crystalline Si solar cells†

Yeasin Arafat Pritom,[a] Dipayon Kumar Sikder,[b] Sameia Zaman,[b,c] and Mainul Hossain*[a]

[a]*Department of Electrical and Electronic Engineering, University of Dhaka, Dhaka-1000, Bangladesh.*
[b]*Department of Electrical and Electronic Engineering, Bangladesh University of Engineering and Technology, Dhaka-1205, Bangladesh.*
[c]*Department of Electrical Engineering and Computer Science, Massachusetts Institute of Technology, Cambridge, MA 02139, USA.*
*E-mail: mainul.eee@du.ac.bd

2. Methodology

2.1 Device structure and simulation method

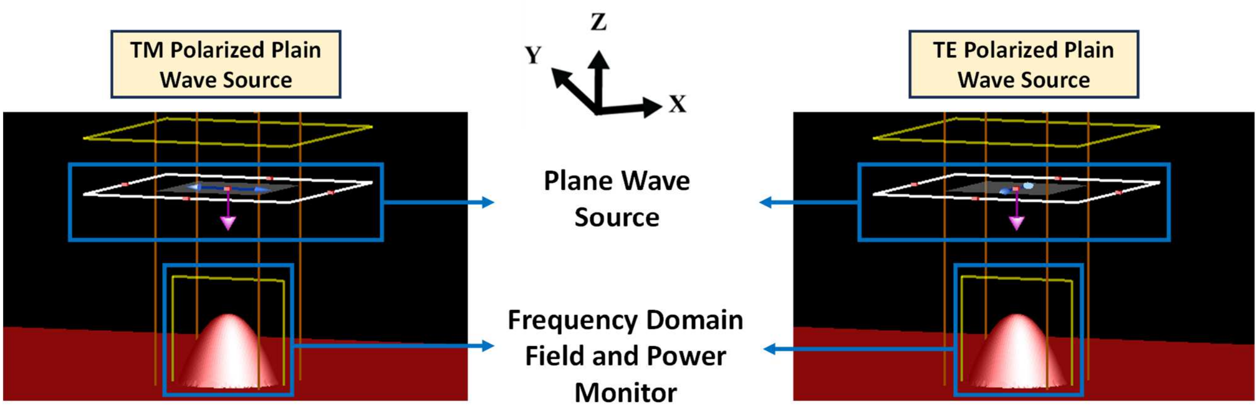

Fig. S1† Simulation setup showing the position of the field and power monitors for both TM and TE polarized incident waves.

3. Result and discussion

3.3 Nanoparticle size

Table S1† Change in filling ratio with diameter of Au NP

Diameter of Au NP (d_{Au})	Base Width of parabola (W_b)	Period (P) $P = W_b + 2d_{Au}$	Height of parabola (H)	Filling Ratio (W_b/P)
Bare Parabola	450 nm	450 nm	500 nm	1.000
20 nm	450 nm	490 nm	500 nm	0.918
25 nm	450 nm	500 nm	500 nm	0.900
30 nm	450 nm	510 nm	500 nm	0.882
35 nm	450 nm	520 nm	500 nm	0.865
37 nm	450 nm	524 nm	500 nm	0.859
39 nm	450 nm	528 nm	500 nm	0.852
40 nm	450 nm	530 nm	500 nm	0.849
42 nm	450 nm	534 nm	500 nm	0.843
45 nm	450 nm	540 nm	500 nm	0.833
50 nm	450 nm	550 nm	500 nm	0.818

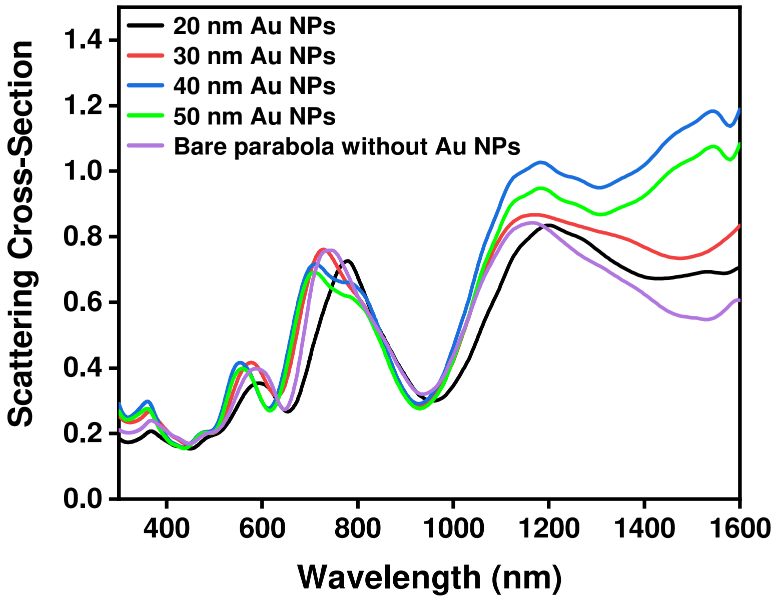

Fig. S2† Results of FDTD simulations with TM polarized incident light showing scattering cross-sections of Au NP embedded Si parabola with different diameters of Au NPs for wavelengths ranging between 300 nm and 1600 nm.

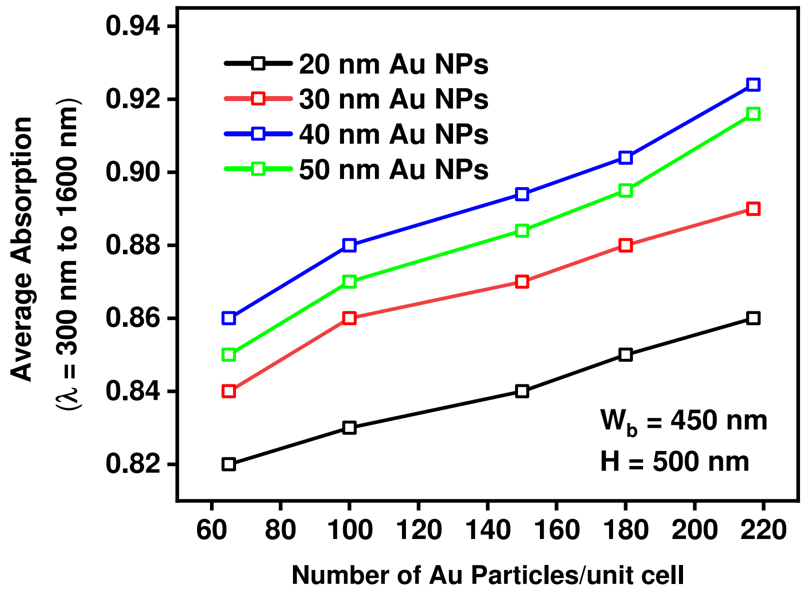

Fig. S3† Results of FDTD simulations with TM polarized incident light showing the average absorption between λ = 300 nm and 1600 nm vs the number of Au NPs per unit cell of the parabola. The diameters of the Au NPs are taken as 20 nm, 30 nm, 40 nm, and 50 nm.

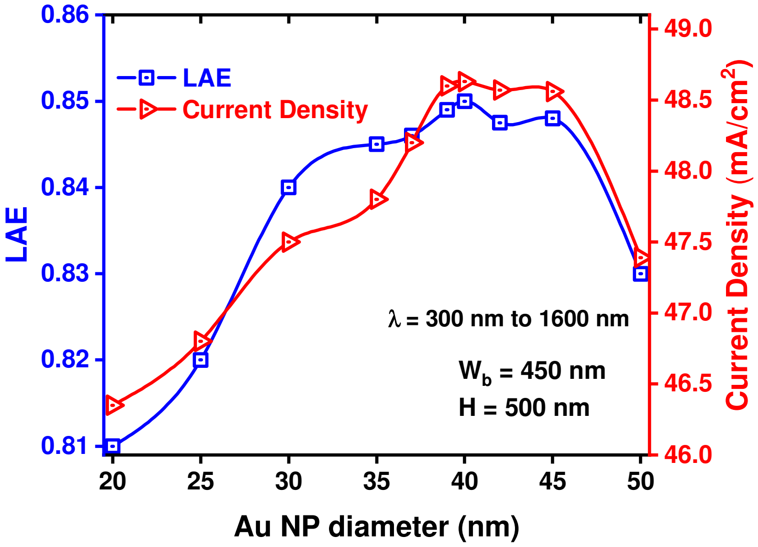

Fig. S4† Results of FDTD simulations with TM polarized incident light showing the light absorption efficiency (LAE)* and current density between λ = 300 nm and 1600 nm for Au NPs having different diameters.

*LAE is calculated as follows:[1]

$$LAE = \frac{\int_{300nm}^{1600nm} \frac{\lambda}{hc} A(\lambda)_{with\_NPs} I_{AM1.5G}(\lambda) d\lambda}{\int_{300nm}^{160nm} \frac{\lambda}{hc} I_{AM1.5G}(\lambda) d\lambda}$$

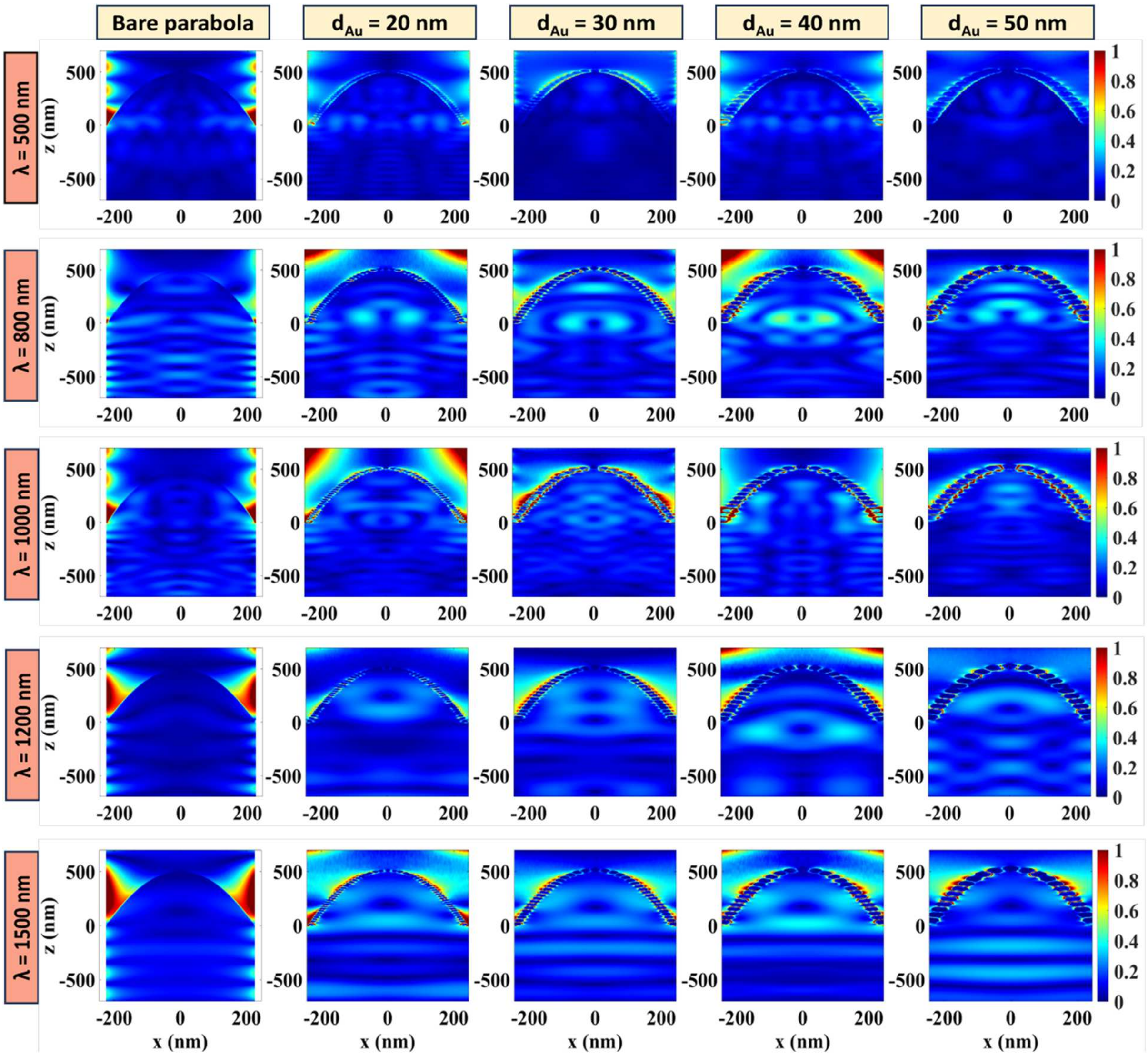

Fig. S5† The normalized electric-field distributions of the bare and Au NP coated parabolic nanostructures for the TE polarized incident light at λ = 500 nm, 800 nm, 1000 nm, 1200 nm, and 1500 nm. The diameters of the Au NPs are taken as 20 nm, 30 nm, 40 nm and 50 nm and bare Si parabola is shown as the reference.

References

1 S. Zaman, M. M. Hassan, M. Hasanuzzaman and M. Z. Baten, *Opt. Express*, 2020, **28**, 25007.
